# Prevalence of antecedent Kawasaki disease in young adults with suspected acute coronary syndrome in high incidence cohort

**DOI:** 10.3389/fcvm.2023.1167771

**Published:** 2023-08-04

**Authors:** Tsung-Cheng Shyu, Chiung-Jen Wu, Yun-Ching Fu, Yi-Chin Peng, Tzu-Yao Chuang, Ho-Chang Kuo, Kai-Sheng Hsieh, I-Hsin Tai

**Affiliations:** ^1^ Department of Pediatric Cardiology, Structural/Congenital Heart Disease and Echocardiography Center, China Medical University Children’s Hospital, Taichung, Taiwan; ^2^ Department of Pediatrics, College of Medicine, China Medical University, Taichung, Taiwan; ^3^ Division of Cardiology, Kaohsiung Chang Gung Memorial Hospital, College of Medicine, Chang Gung University, Kaohsiung, Taiwan; ^4^ Department of Pediatrics, Taichung Veterans General Hospital, Taichung, Taiwan; ^5^ Kawasaki Disease Center and Pediatrics, Kaohsiung Chang Gung Memorial Hospital, College of Medicine, Chang Gung University, Kaohsiung, Taiwan

**Keywords:** Kawasaki disease (KD), prevalence, acute coronary syndrome, coronary aneurysm, low-density lipoprotein, young adult

## Abstract

**Background:**

Acute coronary syndrome (ACS) in early adulthood (<40 years old) may be associated with unrevealed diagnoses of Kawasaki disease (KD) in childhood. Daniels et al. showed that 5% of young adults with acute coronary syndrome might have antecedent Kawasaki disease in a cohort with Kawasaki disease incidence rates ranging from about 9 to 20 per 100,000 children under 5 years of age. However, there is no relevant research from the cohort with higher incidence rates (>80–100 per 100,000 children under 5 years of age) of Kawasaki disease.

**Methods:**

We conducted a multicenter, retrospective study by reviewing medical records and angiographic data from two institutions (middle and southern Taiwan, respectively) of adults <40 years of age who underwent coronary angiography for clinically suspected acute coronary syndrome (2009–2019). Angiographic images were independently analyzed by three cardiologists who were blinded to the medical records. Demographic and laboratory data and risk factors of coronary artery disease were integrated to assess the likelihood of antecedent KD.

**Results:**

All 323 young adults underwent coronary angiography, and 27 had coronary aneurysms. The patients’ clinical and angiographic characteristics were evaluated, and 7.4% had aneurysms likely to be associated with KD. Most subjects were male (23/24), and their low-density lipoprotein (LDL) levels were significantly higher (*p* = 0.028) than those of subjects unlikely to have KD.

**Conclusion:**

This study proposed that the cohort with higher Kawasaki disease incidence rates may have a higher prevalence of young adult ACS associated with antecedent KD. The importance of determining the clinical therapeutic significance of antecedent Kawasaki disease in young adult ACS warrants advanced research. Higher LDL levels may have a long-term cardiovascular impact in KD patients with persistent coronary aneurysms.

## Introduction

Kawasaki disease (KD) is an acute vasculitis that involves multiple systems and occurs mainly in children younger than 5 years old. Children with KD may develop coronary aneurysms if they do not receive a timely initial high dose of intravenous immunoglobulin (IVIG) within 10 days of the onset of KD. A previous study showed that KD may cause chronic inflammation (1), even if the patient's fever subsides after the administration of IVIG; hence, aneurysms may persist (2). Although the first case of KD in Taiwan was diagnosed by Professor Lu in 1976 (3), pediatricians in Taiwan recently became familiar with KD about 5–10 years ago. With the top three incidence rates [around 80 per million (4) children younger than 5 years old] of KD in the world (3, 5, 6), we speculate that many young adults may have had KD in their childhood, which was not diagnosed and treated promptly. According to the natural course of KD, about 20%–30% of untreated KD cases lead to small, medium, and large coronary aneurysms (2). Although small- and medium-sized coronary aneurysms can resolve spontaneously, large coronary aneurysms rarely regress (7, 8). Persistent aneurysms lead to calcification, thrombosis, and organization, which may contribute to coronary stenotic lesions and myocardial ischemia in early adulthood (<40 years of age) (9, 10).

A previous study performed in a cohort with a relatively low incidence of KD (<20 per 100,000 children younger than 5 years old) revealed that about 5% of young adults <40 years of age, who were evaluated using angiography for acute coronary syndrome (ACS), may have had antecedent KD if the angiography disclosed a compatible result, coronary aneurysmal change (11). There are some vital points contributed to the execution of the present study and should be addressed. First, it is widely accepted that KD may be present as a post-infectious state in genetically susceptible individuals, which means that children with different genetic bases would have different incidence rates; hence, we chose a cohort with a high incidence of KD to research. Second, the American Heart Association guidelines defined giant coronary aneurysm associated with KD if the diameter exceeded 8.0 mm, in spite of the fact that small- or medium-sized aneurysms could regress and their persistent presence (7). According to the “JCS/JSCS 2020 Guideline on Diagnosis and Management of Cardiovascular Sequelae in Kawasaki Disease,” the sequelae indicate failure of endothelial function in the coronary aneurysm, whether it regressed or not. Therefore, the previous research methodology may seem to be against the guidelines in some results and in such a setting may have underestimated the true prevalence of the cardiovascular sequelae of KD in young adults because the regressed aneurysm may be undetected. Therefore, we conducted the first young adult ACS investigation in a cohort with a high incidence of KD.

## Methods

### Subjects’ enrollment criteria

The study was approved by the Institutional Review Boards of two medical centers (MC) in Taiwan. The enrollment criteria for the study were as follows:

- Age: Participants must be under 40 years old.
- Medical history: Participants must have a history of suspected acute coronary syndrome.
- Procedure: Participants must have undergone percutaneous coronary intervention (PCI) at the study institution.
- Period: The study period was from January 1, 2009, to December 31, 2019.

The objective of the study was to investigate the prevalence of ACS in this population and identify the factors associated with its occurrence.

### Data acquisition

We assessed patients who were <40 years of age who underwent selective coronary angiography due to suspicion of ACS. The criteria used to identify patients with “suspicion of ACS” in our study were based on clinical symptoms, serial electrocardiogram (ECG), or cardiac enzyme changes. Specifically, patients who presented to the emergency department with chest pain, shortness of breath, nausea, vomiting, or diaphoresis and had serial ECG findings with or without cardiac enzyme elevation were considered to have “suspicion of ACS.” Data pertaining to the patients’ demographic characteristics, medical history, laboratory values, and invasive coronary angiography were reviewed from their medical records. However, these details cannot be disclosed in order to maintain patient confidentiality and adhere to informed consent requirements. Risk factors of coronary artery disease (CAD) were defined as diabetes mellitus, hypertension, hyperlipidemia, current smoking, and a family history of premature CAD. Family history of premature CAD was defined as CAD in a first-degree relative before the age of 55 in males and 60 in females, or a physician-documented positive early family history. Hyperlipidemia was defined as a low-density lipoprotein (LDL) cholesterol level of >160 mg/dl. Hypertension was defined as a physician-documented history of high blood pressure. Diabetes mellitus was defined as physician-documented history or HbA1c exceeding 6.5% (12). The methodology was modified and designed according to the research by Daniels et al. (11).

### Adjudication of cases

After the initial quality check-up of the pooled subjects, those who lacked angiography evidence of aneurysms were excluded. Aneurysms were defined as having an aneurysmal shape (saccular or fusiform) and a diameter that was 1.5 times larger than adjacent segments, as per the criteria set by the Japanese Ministry of Health (13).

Secondary, the selected coronary angiograms were reviewed by three cardiologists who were blinded to the patients’ medical histories. The three cardiologists assessed each coronary artery for the presence of stenosis (location and percentage) and aneurysms (location and size) and made suggestions regarding antecedent KD based on the integrated coronary imaging. The coronary segments angiography was considered positive for KD-associated aneurysm after more than two cardiologists confirmed it.

Furthermore, the authors thoroughly assessed each patient and carefully examined their medical records in order to determine the probability of having antecedent KD. Validation of coronary diameter with a previously established normal distribution database is essential in defining the absolute internal diameter of a coronary aneurysm in individuals with antecedent KD. To address this, Dodge et al. conducted a review of 9,160 consecutive angiographic studies and established a comprehensive normal reference for each coronary segment (14), with a repeat measurement error of 0.12 mm. Following the approach demonstrated by Dodge et al., we considered coronary dilatation to be significant when the internal diameter of the coronary artery exceeded the mean plus 2 SDs. Specifically, values exceeding 6 mm for the left main coronary artery (LMCA), 4.6 mm for the left anterior descending (LAD) artery, 5.1 mm for the right coronary artery (RCA), or 5.4 mm for the left circumflex artery (LCX) were regarded as significant coronary dilatation. The validation results of our subjects were consistent with the presence of antecedent KD and significant coronary dilatation when compared to the normal distribution.

**Figure 1:**
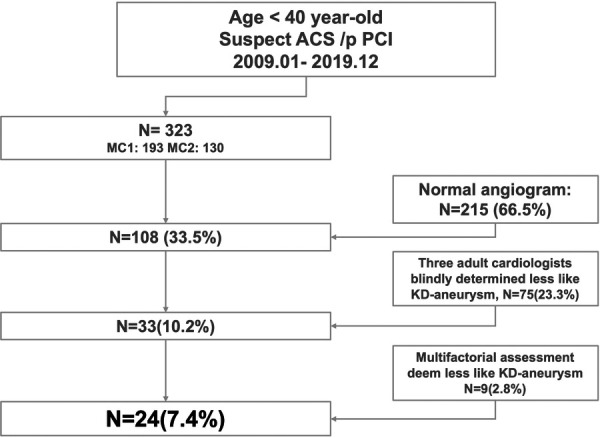
The flowchart illustrating the selection process employed in the current investigation.

Clinical characteristics suggestive of antecedent KD in patients with coronary aneurysms included the absence of risk factors of CAD and age <30 years. Risk factors of CAD were defined as diabetes mellitus, hypertension, hyperlipidemia, current smoking, and a family history of premature CAD. A questionnaire was sent to the subjects with positive aneurysm to confirm the KD-compatible history. Figure 1 shows the flowchart that depicts the sequential steps undertaken to identify and include the appropriate subjects for the study, ensuring a systematic and rigorous approach to participant selection.

### Statistics analysis

All data were expressed as mean and SD, or total numbers and percentages for continuous and categorical variables, respectively. Depending on the normality of the data, the independent *t*-test or Mann–Whitney *U-*test was used to compare differences between two groups of continuous variables. For categorical variables, the *χ*^2^ test or Fisher's exact test was used to determine the difference in frequencies between two groups. A *p*-value of <0.05 was considered statistically significant for all statistical tests. All statistical analyses were performed using SPSS version 19.0 (SPSS Inc., Chicago, IL, USA).

We utilized Cronbach's alpha to test for inter-observer variation in the present research.

## Result

### Characteristics of eligible subjects

Over the 10-year study period (2009–2019), a total of 193 adults (<40 years of age) who underwent coronary angiography at medical center 1 (MC1) met the study criteria. A total of 130 adults at medical center 2 (MC2) were enrolled between 2015 and 2019. The demographic characteristics of the subjects are presented in Table 1. The mean age of the patients was 34.75 years, and 69.3% were male. More than half of the subjects were current smokers, and 17.1% and 27.7% of the subjects had hypertension and diabetes, respectively. Five patients had a known history of KD from medical records. Only 10 subjects agreed to fill the questionnaire, but no subject reported KD-compatible history.

**Table 1:** Demographic data of the suspect antecedent KD subjects vs. control.

	All subjects	Subjects **unlikely** to have antecedent KD	Subjects **likely** to have antecedent KD	*p*
	*N* = 323	*N* = 299	*N* = 24	
Characteristics				
Age (mean ± SD), years	34.75 ± 5.04	34.82 ± 4.99	33.41 ± 5.92	—
Male, *N* (%)	224 (69.3%)	201 (67.2%)	23 (95.8%)	S
Risk factors, *N* (%)				
Current smoking	191 (59.1%)	180 (60.2%)	11 (45.%)	—
Medical history, *N* (%)				—
Diabetes mellitus	—	53 (17.8%)	2 (5.6%)	—
Hypertension	89 (27.7%)	83 (27.7%)	6 (27.8%)	—
Laboratory values (mean ± SD)				
Total cholesterol, mg/dl	191.09 ± 53.59	189.76 ± 53.17	212.00 ± 57.54	—
Triglycerides, mg/dl	223.52 ± 289.05	224.92 ± 297.02	201.53 ± 104.44	—
HDL, mg/dl	38.62 ± 12.04	38.82 ± 12.26	35.40 ± 7.29	—
LDL, mg/dl	115.61 ± 42.16	114.18 ± 41.24	137.28 ± 50.76	S
HbA1c, %	6.63 ± 1.84	6.66 ± 1.88	6.25 ± 1.11	—

S indicates *p* < 0.05.

After reviewing the coronary angiograms, among the 323 subjects who were enrolled, 66.5% had normal coronary angiograms, 33% had stenosis of at least 50%, and 7.4% (*n* = 24) had coronary artery aneurysms (CAAs) likely due to antecedent KD and were selected from the database. A total of 299 patients were allocated to the control group based on a comprehensive assessment of multiple factors, including aneurysm size, location, age, and CAD risk. A review of medical records showed no significant difference in current smoking (*p* = 0.899), diabetes mellitus (*p* = 0.33), or hypertension (*p* = 1.00) between subjects who were unlikely to have antecedent KD and those with confirmed or suspected antecedent KD. Laboratory data showed no significant difference in the total cholesterol (*p* = 0.083), triglyceride (0.747), high-density lipoprotein (HDL) (*p* = 0.186), or HbA1C levels (*p* = 0.958) between subjects who were unlikely to have antecedent KD and subjects with confirmed or suspected antecedent KD. It is worth noting that LDL levels between the two groups were significantly different (*p* = 0.028) as subjects with confirmed or suspected antecedent KD had higher LDL levels compared to subjects who were unlikely to have antecedent KD (Table 1).

### Description of the 24 subjects likely to have antecedent KD

**Figure 2:**
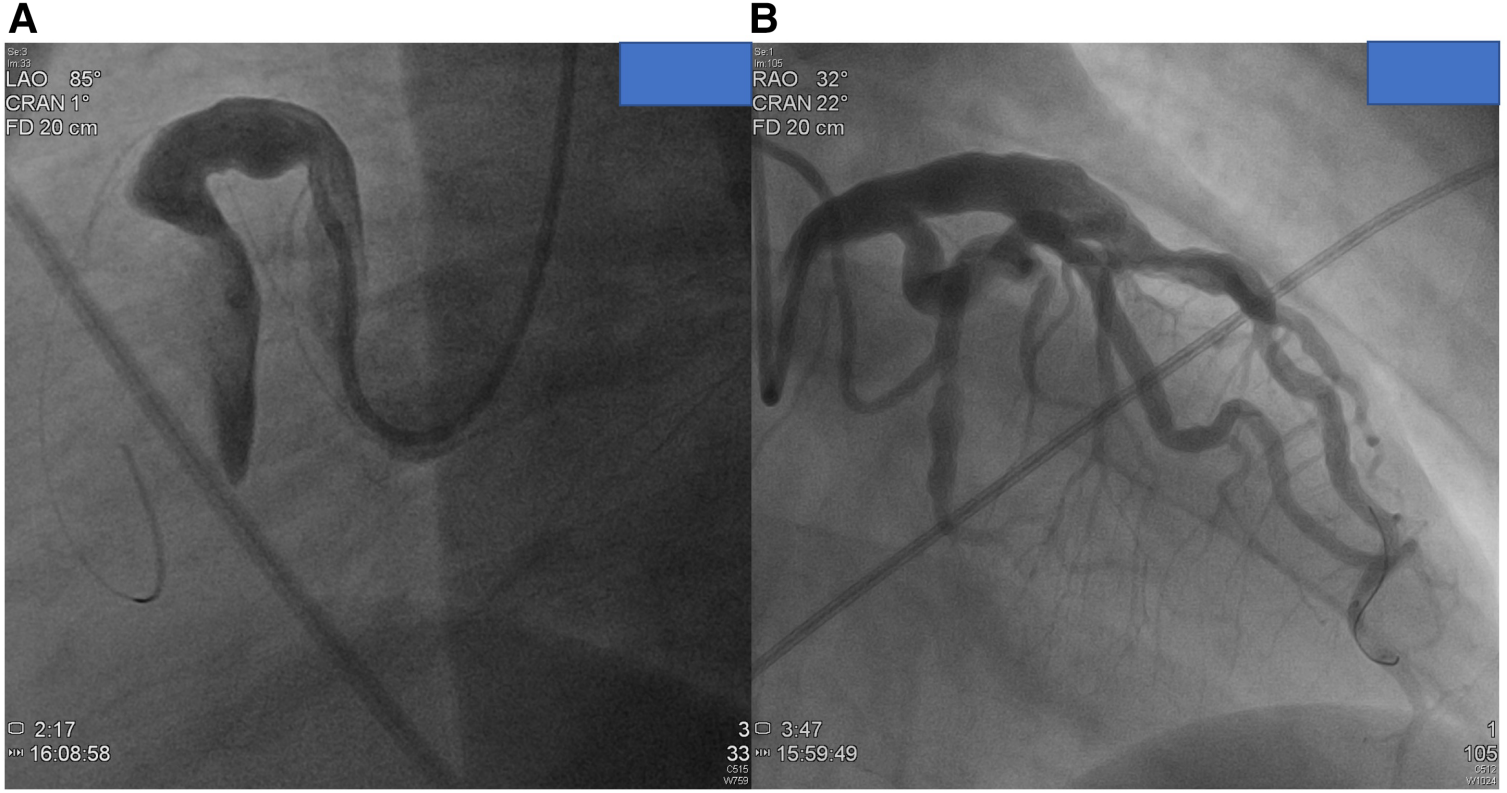
Images from coronary angiograms of patients with highly suspect of antecedent Kawasaki disease. (**A**) A huge fusiform RCA in proximal location from patient 10. (**B**) A diffuse dilated LAD artery with multiple proximal aneurysms over the left circumflex artery from patient 10.

The detailed information of the 24 subjects who were likely to have antecedent KD is presented in Table 2. Among them, patients 1–5 had confirmed medical records of KD and showed different characteristics of their aneurysms. Patients 1 and 3 had persistent aneurysms without significant vascular occlusion, while patient 2 had total occlusion of the left coronary artery (LCA) aneurysm and underwent bare metal stent placement. Patients 4 and 5 also had total occlusion of the corresponding coronary arteries but did not receive stent placement due to sufficient collateral circulation. Serial selective coronary angiography in patient 4 revealed spontaneous revascularization of the occluded coronary segment. Patients 6–14 exhibited coronary aneurysms with an internal diameter exceeding 8.0 mm (Figure 2), regardless of the aneurysm location, which is highly suggestive of KD etiology in the young population. Patients 15–17 had coronary aneurysms over proximal coronary segments, although the aneurysms were less than 8 mm in size. Patients 18–24 showed a lower percentage (42.8% vs. 88.8%) of proximally located aneurysms and smaller aneurysm sizes (6.565 ± 0.612 vs. 11.176 ± 6.33, *p* = 0.04) compared to the other patients in the group. We additionally list the critical difference between the present study and Daniel et al. (Table 3). In this study, we utilized Cronbach's alpha to test for inter-observer variation. The test yielded a strong level of consistency between raters, with a Kappa value of 0.815 (*p* < 0.001), indicating significant agreement between the raters. This statistical analysis was conducted to ensure that the model performance was consistent and reliable.

**Table 2:** Subjects likely to have KD-associated coronary aneurysms, M/F = 23/1.

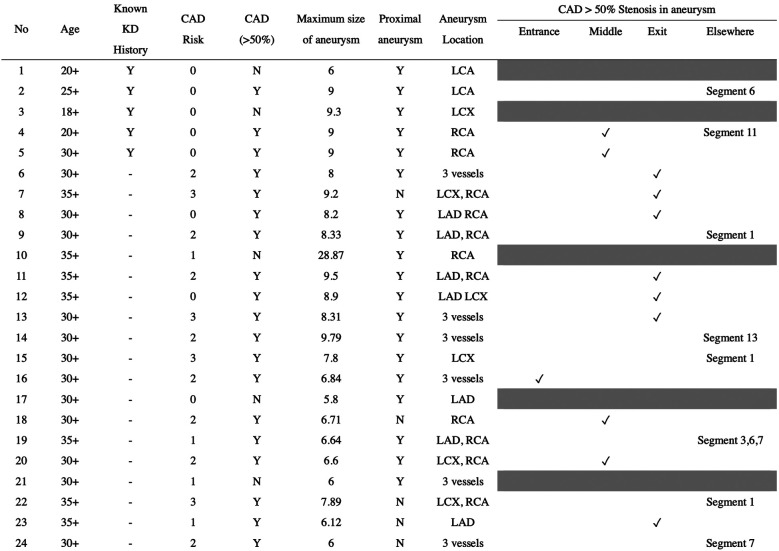

Coronary arterial system comprises 15 segments, including RCA proximal segment (1), medial segment (2), distal segment (3); PDA (4); left main coronary artery (5); LAD proximal segment (6), medial segment (7), apical segment (8), D1 (9), D2 (10); LCX proximal segment (11); OM (12), distal segment (13); left PL (14); and PDA (15).

LCX, left circumflex; Y, yes; N, no; D1, first diagonal branch; D2, second diagonal branch; PL, posterolateral branch; PCA, posterior descending artery; OM, obtuse marginal branch.

**Table 3:** Key point comparison between studies in Taiwan and the US.

	Daniels et al. (2012) (US)	Shyu et al. (2022) (Taiwan)
Cohort annual incidence of KD	20/million	80/million
Total enrolled subjects, *N*	261	323
Definite KD, *N* (decision policy)	4 (History taking)	5 (Medical records)
Likely antecedent KD, *N* (%)	16 (5)	24 (7.4)
LDL (likely vs. unlikely), *p*-value	0.09	0.028

## Discussion

### Different incidence, different prevalence

Numerous research studies have consistently demonstrated an increase in the incidence of KD, particularly in the atypical form (15). It can be associated with severe coronary artery complications, persistent or giant coronary aneurysms (16, 17), if undisclosed timely, to which the pediatrician can ill afford to miss these diagnoses.

Currently, KD has become one of the most popular diseases and is rare for pediatricians to ignore; however, this was not the case in the past. Hence, we speculate that many adults could have silent variable-sized coronary aneurysms secondary to childhood undiagnosed KD, which manifest as ACS in early adulthood two or three decades after the onset of KD.

The prevalence of suspected antecedent KD in young adults with suspected ACS is 5% and 7.4% according to Daniels et al. and the present study (Table 3), respectively. Given its occurrence in genetically susceptible individuals (18), KD is more prevalent in children younger than 5 years old of the present cohort [20 (19) vs. 80 (4) per million children <5 years old]; however, the prevalence difference of antecedent KD is smaller than expected. This may be due to a multi-factor phenomenon, including genetic diversity, medical source accessibility, diet habits, and self-health management awareness (20).

### Differential coronary artery aneurysms

**Figure 3:**
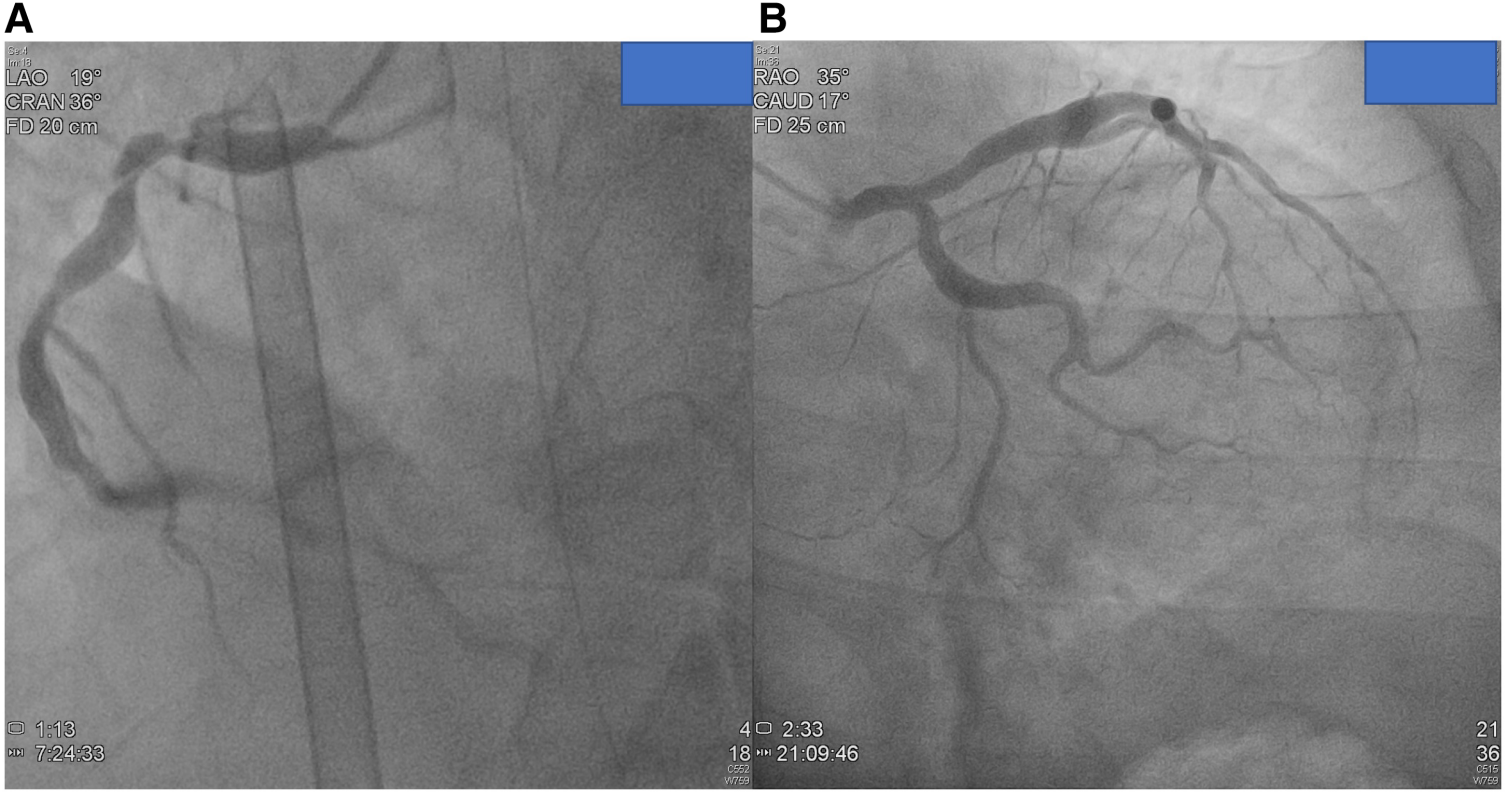
Images from coronary angiograms of patients unlikely have antecedent Kawasaki disease. (**A**) Small coronary aneurysms over proximal and middle RCA without significant stenotic lesion from patient 26. (**B**) Small coronary aneurysm over proximal left circumflex artery and LAD. Evident ectasia in left main coronary artery (patient 25).

CAA is characterized by the dilation of an arterial segment to at least 1.5 times the diameter of the adjacent normal coronary artery. The prevalence of CAA ranges from 0.3% to 4.9% in adult coronary angiographic studies and 0.22% in autopsy series (21). In about 50% of adult cases, CAA is attributed to atherosclerosis, often coexisting with coronary artery disease (Figure 3) (22, 23). Approximately 20%–30% of cases are considered congenital in origin. Inflammatory or connective tissue diseases, such as scleroderma, Ehlers–Danlos syndrome, (Anti-Neutrophil Cytoplasmic Antibodies) ANCA-related vasculitis, syphilitic aortitis, and Kawasaki disease, account for a smaller percentage of CAA cases (23, 24). While some of these conditions can be easily differentiated based on medical records, the prevalence and clinical implications of CAA remain of interest.

Yuhan Qin et al. identified risk factors for hyperlipidemia-associated CAAs, such as sex, BMI, diastolic blood pressure, D-dimer, triglycerides, and the LDL/HDL ratio. Distinctively, their study found that patients with atherosclerosis-related CAA tended to be older (>60 years old), which is in contrast with our cohort (23).

### Hyperlipidemia (low-density lipoprotein cholesterol) may correlate to coronary aneurysms

Although Daniels et al. revealed no significant change in serum LDL cholesterol levels between subjects unlikely to have KD and subjects with definite or presumed KD (*p* = 0.09), our study provided contrary results (*p* = 0.028, Table 3). Low-density lipoprotein receptor-related protein 1 B (LRP1B) is abundantly expressed in the medial layer of the coronary arteries and is involved in endothelial inflammation. Lin et al. used multivariate regression analyses to identify the associations between LRP1B genetic variations and KD patients to reveal that coronary aneurysm formation in KD was significantly associated with the LRP1B genetic variant (*p* = 0.007) (25). This might explain the discrepancy in the outcomes regarding the role of serum LDL cholesterol levels in patients with coronary aneurysms associated with early-onset ACS between the present study and others. Furthermore, our research team published a significant relationship between serum lipoprotein cholesterol levels and children with KD and coronary artery abnormalities in 2009 (26). We show that in children with KD and coronary artery abnormalities, serum LDL cholesterol level has not significantly decreased after the convalescent phase compared with those without coronary artery abnormalities. It seems to imply that LDL cholesterol has no significant difference in childhood KD but may have significant increase in patients with persistent coronary aneurysms grown to adulthood. Further advanced research has been initiated to elucidate this dynamic process of LDL cholesterol.

### The absence of significant coronary stenosis and proximal location of aneurysms is not universal

Though a previously published document (11) suggests the angiographic criteria indicating coronary aneurysms due to KD include the absence of significant coronary artery stenosis (>50%) and proximal location of aneurysms, our results showed that most (3/5; 60%) of the subjects with confirmed antecedent KD had CAD >50%, and distal location aneurysm can occur in KD (13). Although reasonable, the presence of CAD or distal location of the aneurysm and antecedent KD is not mutually exclusive.

In Table 2, among the 19 subjects diagnosed with CAD and stenotic lesions exceeding 50%, a thorough analysis of lesion distribution revealed notable findings. Specifically, seven lesions were identified distally or at the exit of the aneurysm, indicating the potential occurrence of stenosis in this region. Furthermore, four lesions were observed in the middle of the aneurysmal coronary arteries, while one lesion was localized proximally or at the entry of the aneurysm. These findings underscore the diverse locations where stenosis can manifest within the aneurysm structure. Intriguingly, an additional eight subjects exhibited stenotic lesions beyond the confines of the coronary aneurysm, implying an extension of coronary vasculopathy beyond the aneurysm itself. It is worth mentioning that patient 10, who presented with the largest aneurysm measuring 28.87 mm, did not exhibit any stenosis exceeding 50%. Conversely, patient 19, with a relatively smaller aneurysm measuring 6.64 mm, experienced multiple stenotic lesions. These findings highlight that a larger aneurysm does not guarantee future stenosis, emphasizing the complex and multifactorial nature of this condition (27).

### Future perspective

A retrospective study of young adults (<40 years old) who underwent coronary angiography published in 2017 using a similar cohort showed that only 1.2% (3/245) of the enrolled subjects have an autoimmune disease, including one patient with KD (24). Different methodologies with coronary angiography focused on occlusive CAD, sparing the angiographic characteristics of KD aneurysm, may lead to non-identical results. Our investigation may have the limitation of uncertain KD history because the retrospective analysis was hard to trace medical records decades ago. Questionnaire or interview, which count on memory, benefit little. The future study design should focus on prospective comprehensive methodology with uncontrolled variables as little as possible.

## Conclusion

In this retrospective investigation, we observed a potential trend indicating a higher prevalence of suspected antecedent KD in young adults (under 40 years old) experiencing ACS, particularly in populations with a high incidence of KD. In addition, our findings suggest that KD patients with persistent coronary aneurysms are more likely to have hyperlipidemia, specifically elevated levels of LDL. The elevated LDL cholesterol levels observed in KD patients may contribute to the clinical presentation of ACS or non-ACS. These results emphasize the need for further research to deepen our understanding in this area.

## Data Availability

The original contributions presented in the study are included in the article, further inquiries can be directed to the corresponding author.
